# MicroRNA-1224-5p Aggravates Sepsis-Related Acute Lung Injury in Mice

**DOI:** 10.1155/2022/9493710

**Published:** 2022-06-28

**Authors:** Bing Liu, Feng Chen, Ni-Tao Cheng, Zheng Tang, Xian-Guo Wang, Ming Xu

**Affiliations:** ^1^Department of Respiratory and Critical Care Medicine, Zhongnan Hospital of Wuhan University, Wuhan, 430071 Hubei, China; ^2^Department of Anesthesiology, Zhongnan Hospital of Wuhan University, Wuhan, 430071 Hubei, China; ^3^Department of Thoracic Surgery, Zhongnan Hospital of Wuhan University, Wuhan, 430071 Hubei, China

## Abstract

Oxidative stress and inflammation are implicated in the development of sepsis-related acute lung injury (ALI). MicroRNA-1224-5p (miR-1224-5p) plays critical roles in regulating inflammatory response and reactive oxygen species (ROS) production. The present study is aimed at investigating the role and underlying mechanisms of miR-1224-5p in sepsis-related ALI. Mice were intratracheally injected with lipopolysaccharide (LPS, 5 mg/kg) for 12 h to induce sepsis-related ALI. To manipulate miR-1224-5p level, mice were intravenously injected with the agomir, antagomir, or matched controls for 3 consecutive days. Murine peritoneal macrophages were stimulated with LPS (100 ng/mL) for 6 h to further validate the role of miR-1224-5p *in vitro*. To inhibit adenosine 5′-monophosphate-activated protein kinase alpha (AMPK*α*) or peroxisome proliferator activated receptor-gamma (PPAR-*γ*), compound C or GW9662 was used *in vivo* and *in vitro*. We found that miR-1224-5p levels in lungs were elevated by LPS injection, and that the miR-1224-5p antagomir significantly alleviated LPS-induced inflammation, oxidative stress, and ALI in mice. Conversely, the miR-1224-5p agomir aggravated inflammatory response, ROS generation, and pulmonary dysfunction in LPS-treated mice. In addition, the miR-1224-5p antagomir reduced, while the miR-1224-5p agomir aggravated LPS-induced inflammation and oxidative stress in murine peritoneal macrophages. Further findings revealed that miR-1224-5p is directly bound to the 3′-untranslated regions of PPAR-*γ* and subsequently suppressed PPAR-*γ*/AMPK*α* axis, thereby aggravating LPS-induced ALI *in vivo* and *in vitro*. We demonstrate for the first time that endogenous miR-1224-5p is a critical pathogenic factor for inflammation and oxidative damage during LPS-induced ALI through inactivating PPAR-*γ*/AMPK*α* axis. Targeting miR-1224-5p may help to develop novel approaches to treat sepsis-related ALI.

## 1. Introduction

Acute lung injury (ALI) and its severe form, adult respiratory distress syndrome (ARDS), are life-threatening conditions and cause great mortality to clinical patients, especially those in the intensive care unit [1–3]. Sepsis functions as a common cause of ALI and ARDS, and sepsis-related ALI is characterized as unrestrained inflammatory response and excessive reactive oxygen species (ROS) production in lungs [4, 5]. Till now, no effective and specific approaches are available to treat ALI. Adenosine 5′-monophosphate-activated protein kinase alpha (AMPK*α*) is a multifunctional protein kinase and plays critical roles in controlling inflammation and oxidative stress except for energy homeostasis [6–8]. Emerging studies have also identified an indispensable role of AMPK*α* in the pathogenesis of sepsis-related ALI. Accordingly, Jiang et al. recently revealed that AMPK*α* activation significantly suppressed intrapulmonary inflammation and ROS generation, thereby preventing lipopolysaccharide- (LPS-) induced ALI in mice [4]. Peroxisome proliferator activated receptor-gamma (PPAR-*γ*) is known to act not only on glycolipid metabolism but also on inflammation and oxidative stress in various lung diseases [9–11]. Zhang et al. reported that PPAR-*γ* activation could suppress the activity of nuclear factor-kappa B (NF-*κ*B) and subsequently reduced LPS-induced pulmonary inflammation and ALI [12]. In addition, PPAR-*γ* activation also prevented inflammation and oxidative damage in chronic obstructive pulmonary disease [13]. More importantly, PPAR-*γ* knockdown dramatically augmented hyperoxia-induced inflammation, oxidative stress, and ALI in mice [14]. Taken together, these findings define AMPK*α* and PPAR-*γ* as promising therapeutic candidates to treat sepsis-related ALI.

MicroRNAs (miRNAs) are endogenous, single-stranded noncoding RNAs, and are implicated in multiple cellular processes through binding to the 3′-untranslated regions (UTR) of target messenger RNAs (mRNAs) for translational repression or degradation [15–18]. It is estimated that more than 2000 miRNAs are encoded by the human genome, and they regulate about 60% of all mammalian genes. Dysregulated miRNAs have been detected and validated to participate in the progression of sepsis-related ALI. Our recent study showed that miR-762 expression was increased in LPS-treated lungs, and that the miR-762 antagomir significantly reduced inflammation, oxidative stress, and ALI in mice [19]. miR-1224-5p is famous for its role in several human cancers, including the oesophageal squamous cell carcinoma, hepatocellular carcinoma, and osteosarcoma [20–22]. In addition, miR-1224-5p is also involved in regulating inflammatory response and ROS generation. Niu et al. reported that mouse miR-1224-5p was highly expressed in the spleen, kidney, and lung, and that its level was elevated by LPS stimulation [23]. And findings from Cheng et al. revealed that miR-1224-5p was also elevated in L02 hepatocytes by hydrogen peroxide stimulation, and that downregulation of miR-1224-5p significantly alleviated hydrogen peroxide-stimulated apoptosis of hepatocytes [24]. Moreover, Li et al. demonstrated that inhibition of miR-1224-5p dramatically suppressed hypoxia/reoxygenation-induced oxidative stress and apoptosis in H9C2 cardiomyocytes [25]. Based on these data, we herein aim to investigate the role of miR-1224-5p in sepsis-related ALI and also try to validate the involvement of AMPK*α* and PPAR-*γ* in this process.

## 2. Material and Methods

### 2.1. Reagents

LPS from *Escherichia coli* 0111: B4 (L2360) and 2′,7′-dichlorofluorescin diacetate (DCFH-DA, D6883) were purchased from Sigma-Aldrich (St. Louis, MO, USA). Lipid peroxidation assay kit (ab118970), superoxide dismutase (SOD) activity assay kit (ab65354), reduced glutathione (GSH) assay kit (ab235670), mouse interleukin-1 beta (IL-1*β*, ab197742), IL-6 (ab222503), IL-18 (ab216165), tumor necrosis factor-alpha (TNF-*α*, ab208348) ELISA kits, lactate dehydrogenase (LDH) activity assay kit (ab102526), myeloperoxidase (MPO) activity assay kit (ab105136), and caspase-1 activity assay kit (ab39412) were all purchased from Abcam (Cambridge, MA, USA). TransAM® NF-*κ*B p65 kit (40097) was purchased from Active Motif (Carlsbad, CA, USA). Pierce BCA protein assay kit (23225) and NE-PER™ nuclear and cytoplasmic extraction reagent (78835) were purchased from ThermoFisher Scientific (Waltham, MA, USA). Compound C (CpC, S7840) and GW9662 (S2915) were purchased from Selleck Chemicals (Houston, TX, USA). The agomir (miR40005460-4-5), agomir control (AgNC, miR4N0000003-4-5), antagomir (miR30005460-4-5), and antagomir control (AntagNC, miR3N0000003-4-5) of miR-1224-5p were synthesized by RiboBio Co., Ltd. (Guangzhou, China). The following primary antibodies were purchased from Abcam (Cambridge, MA, USA): antinuclear factor E2-related factor 2 (NRF2, ab62352), antinucleotide-binding domain-like receptor protein 3 (NLRP3, ab214185), antithioredoxin interacting protein (TXNIP, ab188865), and anti-PPAR-*γ* (ab209350), while anticapase-1 p10 (sc-56036) was obtained from Santa Cruz Biotechnology (Dallas, Texas, USA). Antiglyceraldehyde-3-phosphate dehydrogenase (GAPDH, 2118S), antiphospho AMPK*α* (P-AMPK*α*, 2535), and antitotal AMPK*α* (t-AMPK*α*, 2603P) were purchased from Cell Signaling Technology (Danvers, MA, USA).

### 2.2. Animals

Bacterial pneumonia is a common cause of sepsis-related ALI, and LPS is the major virulence factor in the outer membrane of Gram-negative bacteria and is well-accepted to generate sepsis-related organ injury in mice. In the present study, one hundred and two male C57BL/6 mice aged 10-12-weeks old received a single intratracheal injection of LPS (5 mg/kg) and then maintained for 12 h to establish sepsis-related ALI as we previously described, while mice with saline injection were used as the control [19, 26]. To manipulate miR-1224-5p level, mice were intravenously injected with the agomir (30 mg/kg/day), antagomir (80 mg/kg/day), or matched controls for 3 consecutive days before LPS injection [19, 26]. To investigate the role of miR-1224-5p in mortality rate, mice were injected from the trachea with a lethal dose of LPS (25 mg/kg) and monitored every 12 h [19]. To inhibit AMPK*α*, mice were intraperitoneally with CpC (20 mg/kg) once two days for 3 times before miR-1224-5p antagomir treatment [4]. To inhibit PPAR-*γ*, mice were pretreated with GW9662 (0.35 mg/kg/day in drinking water) for 10 consecutive days as previously described [27, 28]. The animal protocols were approved by the Animal Experimentation Ethics Committee of Zhongnan Hospital of Wuhan University and were also in accordance with the guidelines of the National Institutes of Health for live animals.

### 2.3. Functional Determination

Pulmonary function was determined using the Buxco pulmonary function testing system (Connecticut, CT, USA) as previously described [26, 29]. Airway resistance, pulmonary ventilation, lung compliance, tidal volume, and respiratory rate (RR) were recorded from the anesthetized mice.

### 2.4. Blood Gas Analysis

Arterial blood gas analysis was performed to evaluate pulmonary gas exchange and blood acid-base status [30]. In brief, arterial blood samples were collected from the right common carotid artery using a heparinized PE10 polyethylene catheter, which were then used to determine the arterial blood gas parameters, including partial pressure of oxygen (PaO_2_), partial pressure of carbon dioxide (PaCO_2_), and sodium bicarbonate (HCO_3_^−^) by an automatic blood gas analyzer.

### 2.5. Lung Wet to Dry Ratio

Lung wet to dry ratio was calculated to evaluate pulmonary edema as we previously described [19, 26]. In brief, fresh lungs were excised and weighed to obtain the wet weight, which were then placed at 80°C for 96 h to get the constant dry weight. Next, the lung wet to dry ratio was calculated.

### 2.6. Bronchoalveolar Lavage Fluid (BALF) Collection and Analysis

The lungs were intratracheally instillated with 1 mL cooled phosphate buffer saline (PBS, pH = 7.4) for 2-3 times to obtain the BALF from a detaining needle inserting to the mouse trachea which was then centrifuged for 10 min at 4°C under a speed of 1600 rpm with the supernatants collected for analysis of cytokines and protein concentrations [31]. Total protein concentrations were measured by the Pierce BCA protein assay kit (ThermoFisher Scientific; Waltham, MA, USA). The pelleted cells were then resuspended in 0.5 mL PBS, and cell numbers were counted with a hemocytometer and the Wright-Giemsa staining as we recently described [19].

### 2.7. Cell Culture and Stimulation

Murine peritoneal macrophages were isolated as previously described [32]. In brief, the peritoneal cavity was rinsed with cooled PBS for 2-3 times to obtain the macrophages-enriched solution, which was then centrifuged for 10 min at 4°C. The pelleted macrophages were then resuspended in RPMI 160 medium and incubated at a density of 2 × 10^6^ per well. To investigate the role of miR-1224-5p *in vitro*, cells were pretreated with the miR-1224-5p agomir (50 nmol/L), antagomir (50 nmol/L), or corresponding controls using Lipo6000™ Transfection Reagent for 24 h, cultured in fresh medium for an additional 24 h, and then stimulated with or without LPS (100 ng/mL) for 6 h [4, 19]. To inhibit AMPK*α* or PPAR-*γ*, cells were preincubated with CpC (20 *μ*mol/L) or GW9662 (10 *μ*mol/L) for 12 h before miR-1224-5p antagomir treatment [4, 27].

### 2.8. Western Blot

Total proteins were extracted as previously described [19, 33–35]. Briefly, fresh lungs or macrophages were lysed with RIPA lysis buffer, and then, total protein concentrations were determined using a commercial kit following the manufacturer's instructions. 20 *μ*g proteins were then separated by SDS-PAGE and transferred onto the PVDF membranes, which were then blocked with 5% BSA for 1.5 h at room temperature and incubated with the indicated primary antibodies overnight at 4°C. After 24 h, the membranes were incubated with horseradish peroxidase-conjugated secondary antibodies for 1 h at room temperature and visualized with enhanced chemiluminescent reagent. Protein levels were quantified to GAPDH or the total proteins using an Image Lab software (Version 6.0).

### 2.9. Quantitative Real-Time PCR

Total RNA from lungs or macrophages were prepared using TRIzol reagent and reversely transcribed to cDNA using the Transcriptor First Strand cDNA Synthesis Kit. Quantitative real-time PCR was then performed using SYBR Green Mix, and relative gene expression was quantified to the internal controls using the 2^-*ΔΔ*Ct^ method [36–39].

### 2.10. Determination of Oxidative Stress

ROS productions were determined using a DCFH-DA probe as previously described [19, 40, 41]. In brief, fresh lung homogenates or macrophages lysates were incubated with DCFH-DA (50 *μ*mol/L) for 30 min at 37°C in the dark, and then, fluorescence intensities were examined at an excitation/emission wavelength of 485/535 nm using a BioTek microplate reader. The levels of malondialdehyde (MDA) content, total SOD activity, and GSH content were measured by commercial kits following the manufacturer's instructions (Abcam; Cambridge, MA, USA).

### 2.11. Biochemical Analyses

Inflammatory cytokines in BALF or lung homogenates were detected by commercial ELISA kits, and LDH activities in lungs were detected at 450 nm using commercial kit. To evaluate NF-*κ*B activities *in vivo* and *in vitro*, nuclear extracts were prepared and incubated with the TransAM® NF-*κ*B p65 kit according to the manufacturer's instructions (Active Motif, Carlsbad, CA, USA) [42]. In addition, fresh lungs were homogenized with MPO assay buffer, centrifuged to remove the insoluble material, and incubated with the reaction mix and TNB reagent/standard solution, which was then detected at 412 nm. Caspase-1 activities were detected using a commercial kit (Abcam; Cambridge, MA, USA) at an excitation/emission wavelength of 400/505 nm based on the fluorescent substrate YVAD-AFC (AFC: 7-amino-4-trifluoromethyl coumarin).

### 2.12. Luciferase Reporter Assay

The PPAR-*γ* 3′-UTR containing a binding site of miR-1224-5p was cloned and inserted into the pGL3 plasmid (Promega; Sunnyvale, CA, USA), which was then transfected to the HEK293T cells for 48 h with or without the miR-1224-5p agomir treatment. Next, cells were lysed, and the luciferase activities were measured by the Dual-Luciferase Reporter Assay System (Promega). Results were calculated as the ratios of *Firefly* luciferase luminescence to *Renilla* luciferase luminescence [43–45].

### 2.13. Statistical Analysis

Comparisons between 2 groups were conducted by an unpaired Student's *t*-test, and differences between more than 2 groups were assessed by one-way ANOVA with Turkey post hoc test for post hoc analysis. Statistical analysis was performed using SPSS software (version 23.0). Data represented as the means ± SD. *P* value less than 0.05 was regarded to be statistically significant.

## 3. Results

### 3.1. The miR-1224-5p Antagomir Attenuates LPS-Induced ALI in Mice

**Figure 1:**
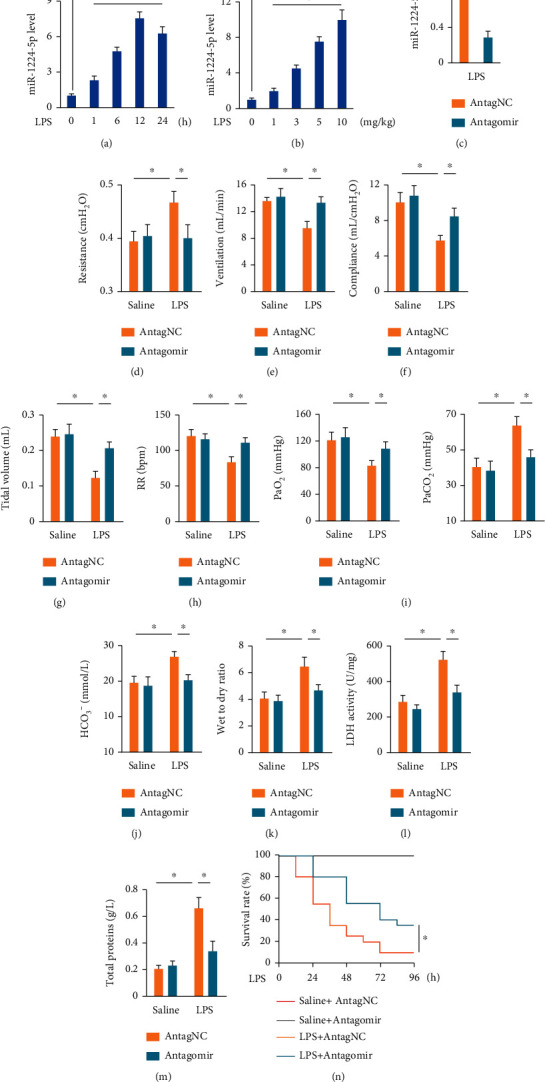
The miR-1224-5p antagomir attenuates LPS-induced ALI in mice. (a) Mice were intratracheally injected with LPS (5 mg/kg), and the levels of miR-1224-5p in lungs were measured at indicating times. (b) Mice were intratracheally injected with indicating doses of LPS for 12 h, and the levels of miR-1224-5p in lungs were measured. (c) Mice were intravenously injected with the miR-1224-5p antagomir (80 mg/kg/day) or AntagNC for 3 consecutive days and then exposed to LPS (5 mg/kg) for 12 h, and the levels of miR-1224-5p in lungs were measured. (d)–(h) Pulmonary function was detected. (i, j) Arterial blood gas analysis of PaO_2_, PaCO_2_, and HCO_3_^−^. (k) Lung wet to dry ratio. (l) LDH activities in lungs. (m) Total protein concentrations in BALF. (n) Mice were intravenously injected with the miR-1224-5p antagomir (80 mg/kg/day) or antagNC for 3 consecutive days and then exposed to a lethal dose of LPS (25 mg/kg), and the survival rate was calculated every 12 h. Data represent the means ± SD (*n* = 6 per group). ∗*P* < 0.05 versus the matched group.

We first evaluated the miR-1224-5p levels in lungs from LPS-treated mice. As shown in Figures 1(a) and 1(b), the miR-1224-5p levels in lungs were elevated by LPS injection in time- and dose-dependent manners. Next, ALI mice were treated with the miR-1224-5p antagomir to inhibit pulmonary miR-1224-5p expression (Figure 1(c)). The results showed that the miR-1224-5p antagomir dramatically reduced airway resistance and increased lung ventilation, compliance, and tidal volume (Figures 1(d)–1(g)). In addition, LPS-induced RR suppression was also improved by the miR-1224-5p (Figure 1(h)). Accordingly, results from arterial blood gas analysis revealed that the miR-1224-5p antagomir significantly increased PaO_2_ and decreased PaCO_2_ and HCO_3_^−^ in ALI mice (Figures 1(i) and 1(j)). LPS-related ALI is commonly accompanied by pulmonary edema. As expected, we observed an increase of lung wet to dry ratio in LPS-treated mice, which was dramatically reduced by the miR-1224-5p antagomir, indicating an ameliorative pulmonary edema (Figure 1(k)). In accordance with the phenotypic alterations, the miR-1224-5p antagomir also inhibited pulmonary injury in ALI mice, as evidenced by the decreased lung LDH activities and total protein concentrations in BALF (Figures 1(l) and 1(m)). Intriguingly, the miR-1224-5p antagomir-treated mice also showed a decreased mortality rate upon LPS stimulation (Figure 1(n)). Taken together, these findings demonstrate that the miR-1224-5p antagomir attenuates LPS-induced ALI in mice.

### 3.2. The miR-1224-5p Agomir Aggravates LPS-Induced ALI in Mice

**Figure 2:**
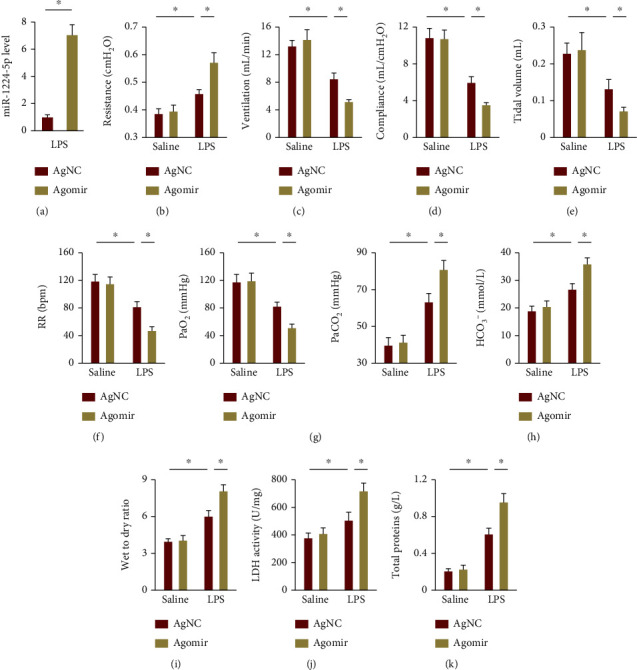
The miR-1224-5p agomir aggravates LPS-induced ALI in mice. (a) Mice were intravenously injected with the miR-1224-5p agomir (30 mg/kg/day) or AgNC for 3 consecutive days and then exposed to LPS (5 mg/kg) for 12 h, and the levels of miR-1224-5p in lungs were measured. (b)–(f) Pulmonary function was detected. (g, h) Arterial blood gas analysis of PaO_2_, PaCO_2_, and HCO_3_^−^. (i) Lung wet to dry ratio. (j) LDH activities in lungs. (k) Total protein concentrations in BALF. Data represent the means ± SD (*n* = 6 per group). ∗*P* < 0.05 versus the matched group.

Next, we treated ALI mice with the miR-1224-5p agomir to overexpress miR-1224-5p in lungs, and the efficiency was presented in Figure 2(a). As shown in Figures 2(b)–2(f), LPS injection resulted in an increase of airway resistance and significant decreases of lung ventilation, compliance, tidal volume, and RR, which were further aggravated by the miR-1224-5p agomir. Meanwhile, the miR-1224-5p agomir further compromised LPS-induced impairment of pulmonary gas exchange and blood acid-base status, as evidenced by the decreased PaO_2_, and increased PaCO_2_ and HCO_3_^−^ in ALI mice (Figures 2(g) and 2(h)). In addition, LPS-induced pulmonary edema and injury were also aggravated in the presence of the miR-1224-5p agomir (Figures 2(i)–2(k)). Furthermore, the miR-1224-5p agomir-treated mice all died within 36 h after LPS injection (data not shown), indicating an increased mortality rate. Altogether, these data reveal that the miR-1224-5p agomir aggravates LPS-induced ALI in mice.

### 3.3. The miR-1224-5p Antagomir Suppresses Oxidative Stress and Inflammation in ALI Mice

**Figure 3:**
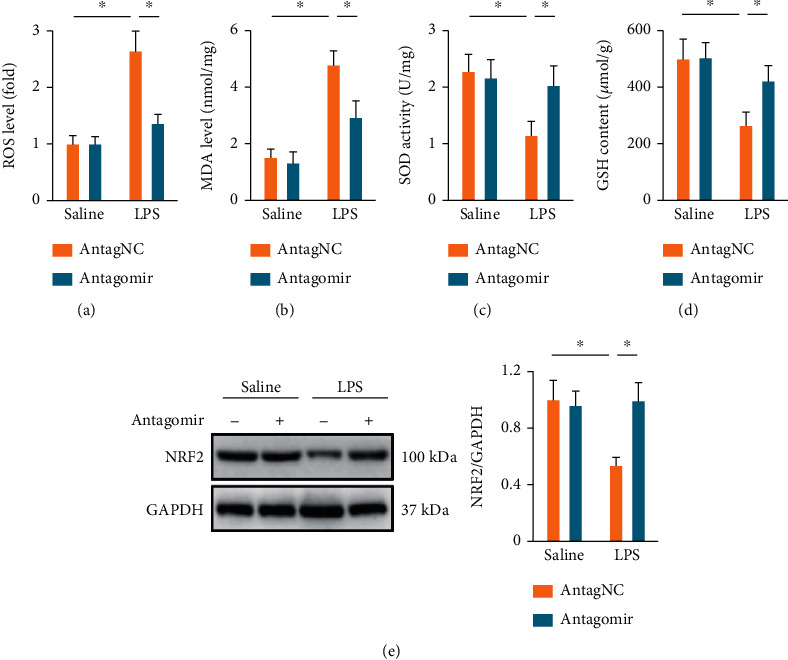
The miR-1224-5p antagomir suppresses oxidative stress in ALI mice. (a) Mice were intravenously injected with the miR-1224-5p antagomir (80 mg/kg/day) or AntagNC for 3 consecutive days and then exposed to LPS (5 mg/kg) for 12 h, and ROS levels in lungs were measured by a DCFH-DA probe. (b) MDA generations in lungs. (c, d) Total SOD activities and GSH content in lungs. (e) Protein levels of NRF2 in lungs. Data represent the means ± SD (*n* = 6 per group). ∗*P* < 0.05 versus the matched group.

**Figure 4:**
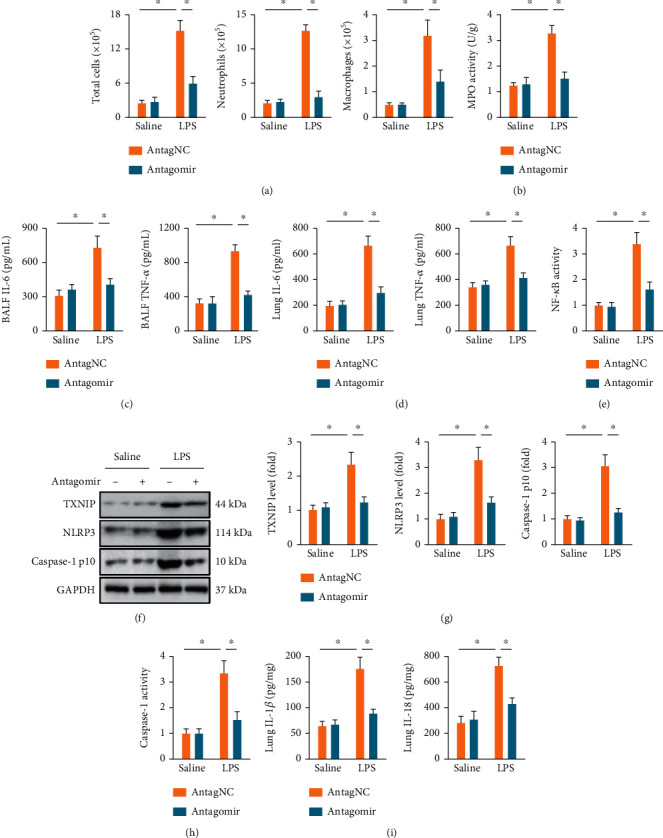
The miR-1224-5p antagomir suppresses inflammation in ALI mice. (a) Mice were intravenously injected with the miR-1224-5p antagomir (80 mg/kg/day) or AntagNC for 3 consecutive days and then exposed to LPS (5 mg/kg) for 12 h. BALF was collected and used to measure the cell numbers. (b) MPO activities in lungs. (c, d) The levels of IL-6 and TNF-*α* in BALF and murine lungs. (e) NF-*κ*B activities in lungs. (f, g) Protein levels of TXNIP, NLRP3 and caspase-1 p10 in lungs. (h) Relative caspase-1 activities in lungs. (i) The levels of IL-1*β* and IL-18 in murine lungs. Data represent the means ± SD (*n* = 6 per group). ∗*P* < 0.05 versus the matched group.

Oxidative stress and inflammation play critical roles in the progression of LPS-induced ALI; therefore, we next evaluated the role of miR-1224-5p in these pathophysiological processes. As shown in Figure 3(a), LPS injection dramatically increased intrapulmonary ROS generation, which was suppressed by the miR-1224-5p antagomir. Accordingly, MDA levels, a product of lipid peroxidation, were also reduced in the miR-1224-5p antagomir-treated ALI mice (Figure 3(b)). SOD and GSH are required to overcome the increased oxidative stress [32]. We found that the miR-1224-5p antagomir dramatically reduced LPS-induced SOD and GSH depletions (Figures 3(c) and 3(d)). Due to the pivotal role of NRF2 in controlling the transcription of these antioxidant enzymes, we evaluated whether the miR-1224-5p antagomir could affect NRF2 pathway. As shown in Figure 3(e), the miR-1224-5p antagomir effectively enhanced the protein expression of NRF2 in LPS-induced ALI mice. Next, we investigated the effects of the miR-1224-5p antagomir on LPS-induced intrapulmonary inflammatory responses in mice. Our findings demonstrated that the miR-1224-5p antagomir dramatically suppressed the accumulation of total cells, neutrophils, and macrophages in BALF (Figure 4(a)). Meanwhile, the activities of pulmonary MPO, an index of neutrophil accumulation, were also inhibited by the miR-1224-5p antagomir (Figure 4(b)). In addition, we found that the miR-1224-5p antagomir effectively reduced the levels of IL-6 and TNF-*α* in lungs and BALF upon LPS treatment (Figures 4(c) and 4(d)). NF-*κ*B functions as a critical molecule to drive the transcription of these inflammatory cytokines [46, 47]. As shown in Figure 4(e), LPS-induced increases of NF-*κ*B activities in lungs were dramatically suppressed by the miR-1224-5p antagomir. Recent findings have identified an indispensable role of NLRP3 inflammasome in LPS-induced inflammation and ALI; therefore, we next investigated NLRP3 inflammasome activation. Upon ROS stimulation, TXNIP detaches from thioredoxin, binds to NLRP3, and then activates NLRP3 inflammasome to amplify inflammatory response [48, 49]. Consistent with the suppressed oxidative stress, we found that the miR-1224-5p antagomir significantly reduced TXNIP expression. Meanwhile, the protein levels of NLRP3 and caspase-1 p10 were also decreased by the miR-1224-5p antagomir, accompanied by a decreased caspase-1 activities in lungs from ALI mice (Figures 4(f)–4(h)). Activation of NLRP3 inflammasome governs the processing and release of proinflammatory cytokines, such as IL-1*β* and IL-18 [32]. As expected, the miR-1224-5p antagomir significantly reduced LPS-induced increases of IL-1*β* and IL-18 in lungs (Figure 4(i)). Collectively, our results prove that the miR-1224-5p antagomir suppresses oxidative stress and inflammation in ALI mice.

### 3.4. The miR-1224-5p Agomir Exacerbates Oxidative Stress and Inflammation in ALI Mice

We investigated whether the miR-1224-5p agomir could aggravate LPS-induced oxidative stress and inflammation in lungs. As shown in Figure S1A-B, LPS-induced ROS and MDA formations were further increased in mice treated with the miR-1224-5p agomir. In addition, the miR-1224-5p agomir also suppressed LPS-induced infiltrations of inflammatory cells (Figure S1C-D). Accordingly, expressions of the inflammatory cytokines, including IL-6, TNF-*α*, IL-1*β*, and IL-18, in LPS-injured lungs were all increased by the miR-1224-5p agomir. Together, we clarify that the miR-1224-5p agomir exacerbates oxidative stress and inflammation in ALI mice.

### 3.5. The miR-1224-5p Antagomir Alleviates, while the miR-1224-5p Agomir Aggravates Oxidative Stress and Inflammation in LPS-Stimulated Macrophages

**Figure 5:**
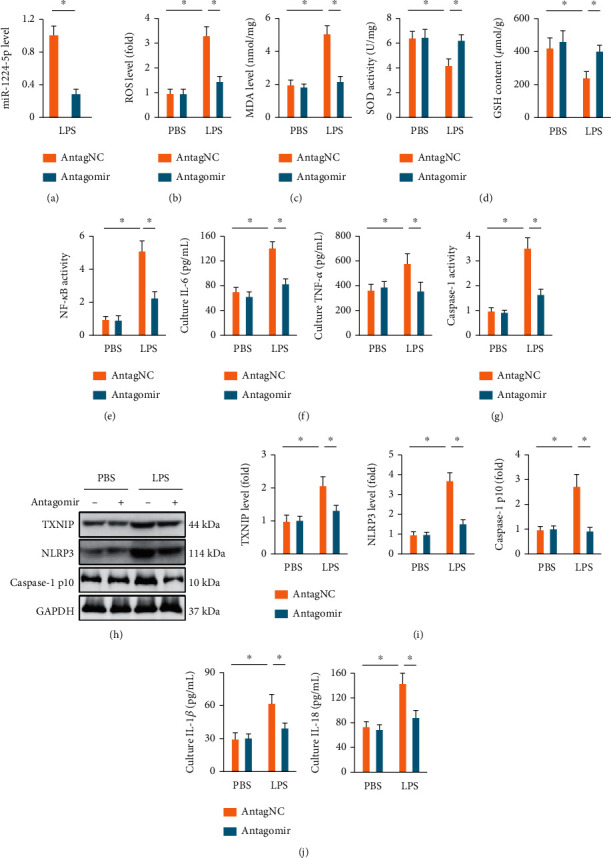
The miR-1224-5p antagomir alleviates oxidative stress and inflammation in LPS-stimulated macrophages. (a) Macrophages were pretreated with the miR-1224-5p antagomir (50 nmol/L) for 24 h, cultured in fresh medium for an additional 24 h, and then stimulated with or without LPS (100 ng/mL) for 6 h. The levels of miR-1224-5p were detected. (b) ROS levels in macrophages were measured by a DCFH-DA probe. (c) MDA generations in macrophages. (d) Total SOD activities and GSH content in macrophages. (e) NF-*κ*B activities in macrophages. (f) The levels of IL-6 and TNF-*α* in the culture of macrophages. (g) Relative caspase-1 activities in macrophages. (h, i) Protein levels of TXNIP, NLRP3, and caspase-1 p10 in macrophages. (j) The levels of IL-1*β* and IL-18 in the culture of macrophages. Data represent the means ± SD (*n* = 6 per group). ∗*P* < 0.05 versus the matched group.

Macrophages are a critical cell type in regulating LPS-induced ALI; therefore, we then investigated the role of miR-1224-5p in LPS-induced oxidative stress and inflammation in macrophages. As shown in Figure 5(a), the miR-1224-5p antagomir significantly reduced miR-1224-5p expressions in LPS-stimulated macrophages. In line with the *in vivo* data, we demonstrated that LPS-induced ROS and MDA generations were significantly reduced by the miR-1224-5p antagomir (Figures 5(b) and 5(c)). And the suppressions of total SOD activities and GSH contents caused by LPS were also restored by the miR-1224-5p antagomir in macrophages (Figure 5(d)). Meanwhile, the miR-1224-5p antagomir also significantly decreased the levels of NF-*κ*B activities and IL-6 and TNF-*α* releases (Figures 5(e) and 5(f)). Intriguingly, the activation of NLRP3 inflammasome in LPS-treated macrophages was dramatically suppressed by the miR-1224-5p antagomir, as evidenced by the decreased caspase-1 activities, and TXNIP, NLRP3, and caspase-1 p10 protein levels (Figures 5(g)–5(i)). Meanwhile, the releases of IL-1*β* and IL-18 from LPS-treated macrophages were also inhibited by the miR-1224-5p antagomir (Figure 5(j)). Conversely, LPS-stimulated macrophages were treated with the miR-1224-5p agomir to overexpress miR-1224-5p expressions *in vitro* (Figure S2A). As shown in Figure S2B-C, the miR-1224-5p agomir further promoted LPS-induced oxidative stress in macrophages. And the inflammatory response caused by LPS was also amplified by the miR-1224-5p agomir, as evidenced by the increased NF-*κ*B activities, and IL-6, TNF-*α*, IL-1*β*, and IL-18 releases (Figure S2D-F). Collectively, the data imply that the miR-1224-5p antagomir alleviates, while the miR-1224-5p agomir aggravates oxidative stress and inflammation in LPS-stimulated macrophages.

### 3.6. The miR-1224-5p Antagomir Prevents LPS-Induced ALI through Activating AMPK*α*

**Figure 6:**
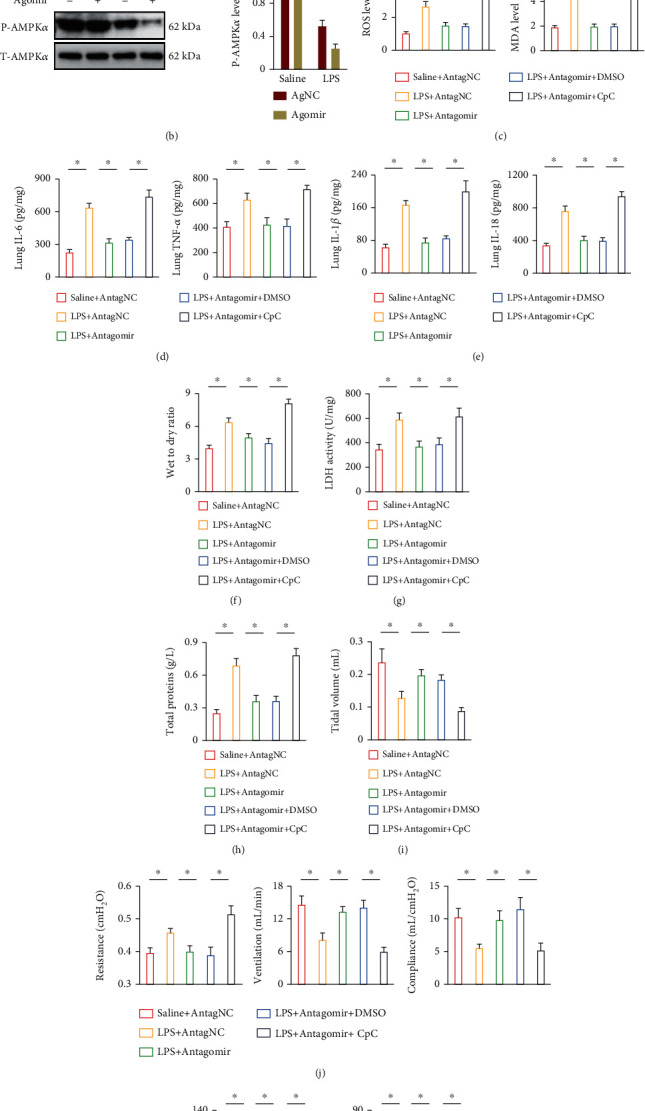
The miR-1224-5p antagomir prevents LPS-induced ALI through activating AMPK*α*. (a, b) Mice were intravenously injected with the agomir (30 mg/kg/day), antagomir (80 mg/kg/day), or matched controls for 3 consecutive days and then exposed to LPS (5 mg/kg) for 12 h. Phosphorylated and total AMPK*α* in lungs were detected. (c) To inhibit AMPK*α*, mice were intraperitoneally with CpC (20 mg/kg) once two days for 3 times before miR-1224-5p antagomir treatment, and then ROS and MDA levels in lungs were measured. (d, e) The levels of IL-1*β*, IL-6, IL-18, and TNF-*α* in murine lungs. (f) Lung wet to dry ratio. (g) LDH activities in lungs. (h) Total protein concentrations in BALF. (i, j) Pulmonary function was detected. (k) Arterial blood gas analysis of PaO_2_ and PaCO_2_. Data represent the means ± SD (*n* = 6 per group). ∗*P* < 0.05 versus the matched group.

Due to the antioxidant and anti-inflammatory effects of AMPK*α* during LPS-induced ALI, we next investigated whether it was involved in the miR-1224-5p antagomir-mediated pulmoprotection against LPS-induced ALI. As shown in Figures 6(a) and 6(b), AMPK*α* phosphorylation in lungs was significantly suppressed by LPS injection, which was preserved by the miR-1224-5p antagomir, but further aggravated by the miR-1224-5p agomir. To validate the necessity of AMPK*α*, mice were pretreated with CpC to inhibit AMPK*α* as previously described. As shown in Figure 6(c), CpC pretreatment significantly abrogated the miR-1224-5p antagomir-mediated antioxidant effects during LPS-induced ALI. In addition, the miR-1224-5p antagomir-mediated inhibitions on IL-6, TNF-*α*, IL-1*β*, and IL-18 were also blocked by CpC (Figures 6(d) and 6(e)). Accordingly, the miR-1224-5p antagomir failed to attenuate LPS-induced pulmonary edema and injury in the presence of CpC, as evidenced by the increased lung wet to dry ratio, LDH activities, and total proteins in BALF (Figures 6(f)–6(h)). The improved pulmonary function and blood gas exchange status by the miR-1224-5p antagomir were also aggravated in CpC-treated mice upon LPS stimulation (Figures 6(i)–6(k)). In line with the *in vitro* data, the antioxidant and anti-inflammatory effects of the miR-1224-5p antagomir were abolished by CpC in LPS-treated macrophages (Figure S7A-D). These findings show that the miR-1224-5p antagomir prevents LPS-induced ALI through activating AMPK*α*.

### 3.7. miR-1224-5p Directly Targets PPAR-*γ* to Regulate AMPK*α* Activation

**Figure 7:**
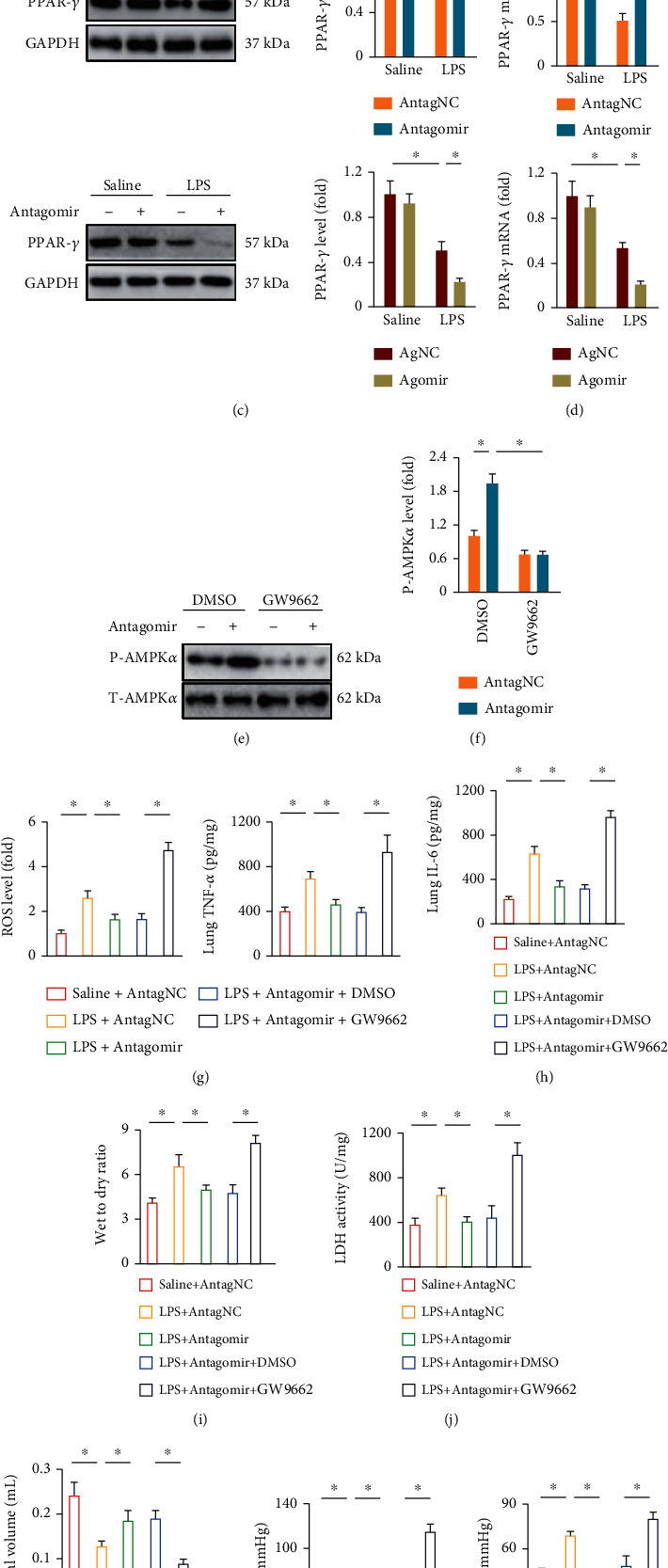
miR-1224-5p directly targets PPAR-*γ* to regulate AMPK*α* activation. (a) The predicted miR-1224-5p-binding site within the PPAR-*γ* 3′-UTR. (b) The PPAR-*γ* 3′-UTR luciferase reporter plasmid was transfected to the HEK293T cells for 48 h with or without the miR-1224-5p agomir treatment. Next, cells were lysed and the luciferase activities were measured. (c, d) Mice were intravenously injected with the agomir (30 mg/kg/day), antagomir (80 mg/kg/day), or matched controls for 3 consecutive days and then exposed to LPS (5 mg/kg) for 12 h. PPAR-*γ* protein and mRNA levels in lungs were detected. (e, f) To inhibit PPAR-*γ*, mice were pretreated with GW9662 (0.35 mg/kg/day in drinking water) for 10 consecutive days, and then, phosphorylated and total AMPK*α* in lungs were detected. (g) ROS levels in lungs were measured. (h) The levels of IL-6 and TNF-*α* in murine lungs. (i) Lung wet to dry ratio. (j) LDH activities in lungs. (k) Tidal volume was detected. (l) Arterial blood gas analysis of PaO_2_ and PaCO_2_. Data represent the means ± SD (*n* = 6 per group). ∗*P* < 0.05 versus the matched group.

We finally explored the potential targets of miR-1224-5p to regulate AMPK*α* activation during LPS-induced ALI. Using the online TargetScan Mouse 7.2 software (http://www.targetscan.org/mmu_72/), we observed a putative binding site of miR-1224-5p on the 3′-UTR of PPAR-*γ*, a nonclassic upstream activator of AMPK*α* (Figure 7(a)). To validate the direct interaction, a luciferase reporter assay was performed. As shown in Figure 7(b), the miR-1224-5p agomir significantly inhibited the luciferase activity of PPAR-*γ* 3′-UTR reporter plasmid. In addition, we demonstrated that the miR-1224-5p antagomir increased, while the miR-1224-5p agomir decreased PPAR-*γ* protein and mRNA levels in lungs from ALI mice (Figures 7(c) and 7(d)). To validate the necessity of PPAR-*γ* in mediating AMPK*α* activation by the miR-1224-5p antagomir, LPS-treated mice were injected with GW9662 to inhibit endogenous PPAR-*γ*. As shown in Figures 7(e) and 7(f), PPAR-*γ* inhibition dramatically abrogated the miR-1224-5p antagomir-induced activation of AMPK*α* in ALI mice. Consistently, the antioxidant and anti-inflammatory effects of the miR-1224-5p antagomir were blocked in GW9662-treated mice upon LPS stimulation (Figures 7(g) and 7(h)). Furthermore, the decreased pulmonary edema, injury, and dysfunction in the miR-1224-5p antagomir-treated ALI mice were also blunted by GW9662 (Figures 7(i)–7(l)). In line with the *in vivo* data, GW9662 also abolished the miR-1224-5p antagomir-mediated antioxidant and anti-inflammatory effects in LPS-treated macrophages (Figure S3A-D). Taken together, our results indicate that miR-1224-5p directly targets PPAR-*γ* to regulate AMPK*α* activation.

## 4. Discussion

ALI and ARDS are life-threatening lung diseases that closely correlate with multiple organ failure and high mortality rate among patients in the intensive care unit. Infectious etiologies are the common causes of ALI, especially the bacterial pneumonia [1]. LPS is a primary component of the outer membrane in Gram-negative bacteria, and it functions as a natural ligand of toll-like receptors (TLRs) to activate the downstream proinflammatory signaling cascades [50, 51]. Previous studies have shown that LPS injection from the trachea caused a severe lung injury that characterized by uncontrolled oxidative stress and inflammatory response, phenocopying the sepsis-related ALI in human [4, 19]. Herein, we used LPS injection to establish sepsis-related ALI in mice. Our findings revealed that the miR-1224-5p levels in lungs were dramatically elevated by LPS injection in time- and dose-dependent manners, and that the miR-1224-5p antagomir could alleviate LPS-induced oxidative stress and inflammation *in vivo* and *in vitro*, thereby preventing ALI progression. Mechanistically, miR-1224-5p directly bounds to and inhibited PPAR-*γ* expression and subsequently suppressed AMPK*α* activation-mediated antioxidant and anti-inflammatory effects. In this study, for the first time, we identify an involvement of miR-1224-5p in the progression of LPS-induced oxidative stress, inflammation, and pulmonary dysfunction, and inhibiting miR-1224-5p may help to develop novel therapeutic approaches to attenuate sepsis-related ALI.

AMPK*α* is well known as an energy sensor in eucaryotic cells and plays critical roles in controlling systemic metabolic homeostasis [52, 53]. However, emerging studies have revealed that AMPK*α* is also implicated in regulating oxidative stress and inflammation. Hu et al. previously reported that AMPK*α* activation by matrine could increase uncoupling protein 2 expression and subsequently reduce doxorubicin-induced ROS overproduction in the heart [54]. In addition, AMPK*α* activation also promoted the expression and nuclear localization of NRF2, which in turn drives the transcription of downstream antioxidant enzymes, such as SOD and GSH [4, 32]. In the present study, we demonstrated that the miR-1224-5p antagomir dramatically suppressed LPS-induced ROS overproduction through activating AMPK*α*, and that CpC treatment abolished the miR-1224-5p antagomir-mediated antioxidant effects *in vivo* and *in vitro*. Inflammation is another feature of sepsis-related ALI. LPS injection directly activates TLR4 and subsequently promoted the phosphorylation and nuclear accumulation of NF-*κ*B, which then drives the transcription of various inflammatory cytokines. The NLRP3 inflammasome functions as a molecular scaffold for the maturation and release of IL-1*β* and IL-18 [55–57]. LPS-triggered ROS generation promotes the dissociation of TXNIP from thioredoxin, which then interacts with NLRP3 to activate NLRP3 inflammasome. Moreover, NF-*κ*B nuclear accumulation also enhances the transcription of NLRP3 inflammasome components. Activation of NLRP3 inflammasome accelerates the processing and release of multiple inflammatory cytokines that in turn further amplify the inflammatory response cascades. AMPK*α* activation has been demonstrated to suppress the activation of NF-*κ*B and NLRP3 inflammasome and reduces the levels of IL-1*β*, IL-6, IL-18, and TNF-*α* [4, 32]. Accordingly, we herein also showed that CpC treatment blocked the miR-1224-5p antagomir-mediated anti-inflammatory effects *in vivo* and *in vitro*. Various classic upstream targets of AMPK*α* have been identified, such as liver kinase B1, calcium/calmodulin dependent protein kinase kinase 2, and protein kinase A [58]. However, using the online TargetScan Mouse 7.2 software, we identified a potential binding site of miR-1224-5p in PPAR-*γ*, a nonclassic upstream activator of AMPK*α*. Previously, Zhang et al. demonstrated that PPAR-*γ* upregulation by rosmarinic acid significantly increased AMPK*α* phosphorylation and activity, and that PPAR-*γ* silence could block rosmarinic acid-mediated AMPK*α* activation [59]. Consistently, we herein showed that PPAR-*γ* was essential for AMPK*α* activation by the miR-1224-5p antagomir. It is worth noting that sepsis-related ALI was generated by LPS injection in this study, a condition unlike the bacterial sepsis-induced ALI to some extent. So the exact role of miR-1224-5p and possible involvement of PPAR-*γ*/AMPK*α* axis in sepsis-related ALI should be further validated in a more physiologically relevant condition (e.g., cecal ligation and puncture models).

In conclusion, for the first time, we demonstrated that miR-1224-5p is implicated in the pathogenesis of LPS-induced oxidative stress, inflammation, and pulmonary dysfunction through inactivating PPAR-*γ*/AMPK*α* axis, and that targeting miR-1224-5p may help to establish novel therapeutic approaches to treat sepsis-related ALI.

## Data Availability

The data that support the findings of this study are available from the corresponding author upon reasonable request.

## Supplementary Materials

Supplementary Materials Figure S1: the miR-1224-5p agomir exacerbates oxidative stress and inflammation in ALI mice. Figure S2: the miR-1224-5p agomir aggravates oxidative stress and inflammation in LPS-stimulated macrophages. Figure S3: miR-1224-5p directly targets PPAR-*γ* to regulate AMPK*α* activation in macrophages.
