# Supplementary material for: MicroRNA-1224-5p Aggravates Sepsis-Related Acute Lung Injury in Mice
Source: Oxid Med Cell Longev. 2022 Jun 28;2022:9493710. doi: 10.1155/2022/9493710 (PMC9256451; doi:10.1155/2022/9493710)
Supplement: Supplementary Materials — Figure S1: the miR-1224-5p agomir exacerbates oxidative stress and inflammation in ALI mice. Figure S2: the miR-1224-5p agomir aggravates oxidative stress and inflammation in LPS-stimulated macrophages. Figure S3: miR-1224-5p directly targets PPAR-γ to regulate AMPKα activation in macrophages. [file 9493710.f1.docx]

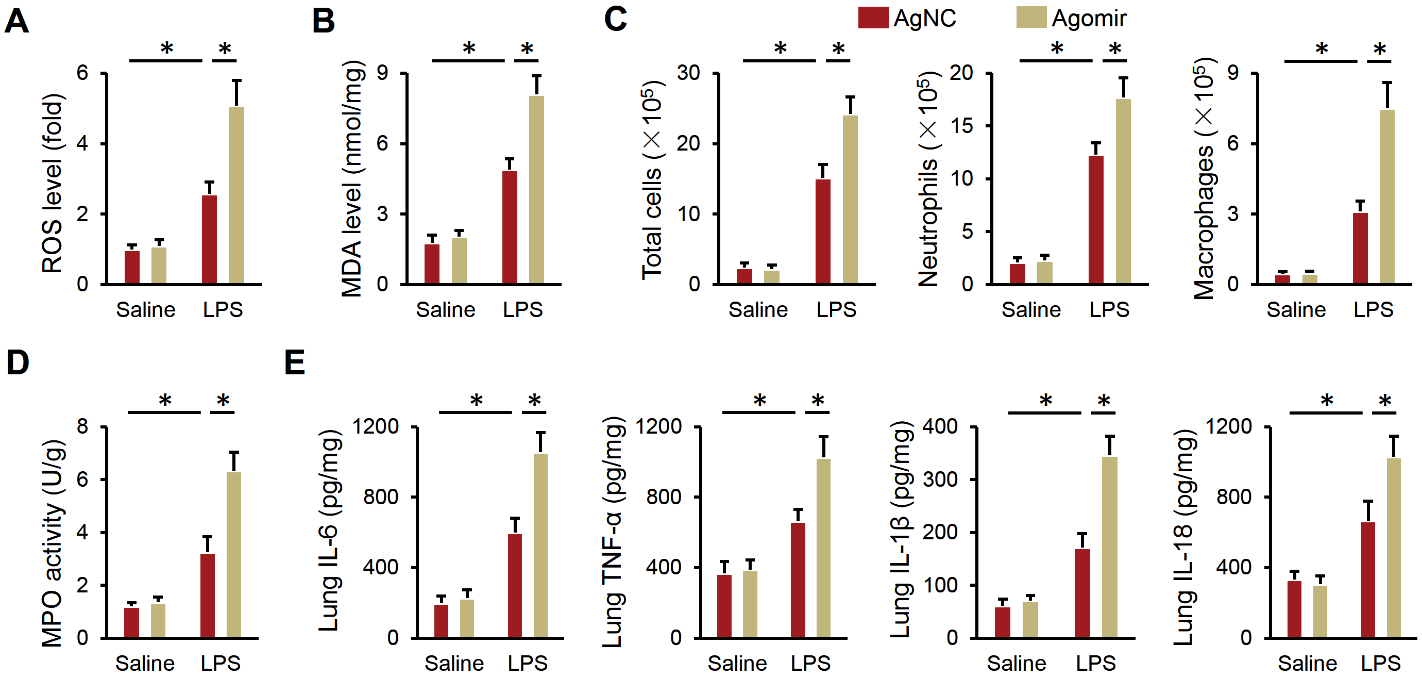


**Figure S1.** **The miR-1224-5p agomir exacerbates oxidative stress and inflammation in ALI mice. (A)** Mice were intravenously injected with the miR-1224-5p agomir (30 mg/kg/day) or AgNC for 3 consecutive days and then exposed to LPS (5 mg/kg) for 12 h, and ROS levels in lungs were measured by a DCFH-DA probe. **(B)** MDA generations in lungs. **(C)** BALF was collected and used to measure the cell numbers. **(D)** MPO activities in lungs. **(C-D)** The levels of IL-6 and TNF-α in BALF and murine lungs. **(E)** The levels of IL-1β, IL-6, IL-18 and TNF-α in murine lungs. Data represent the means ± SD (n = 6 per group). **P* < 0.05 versus the matched group.


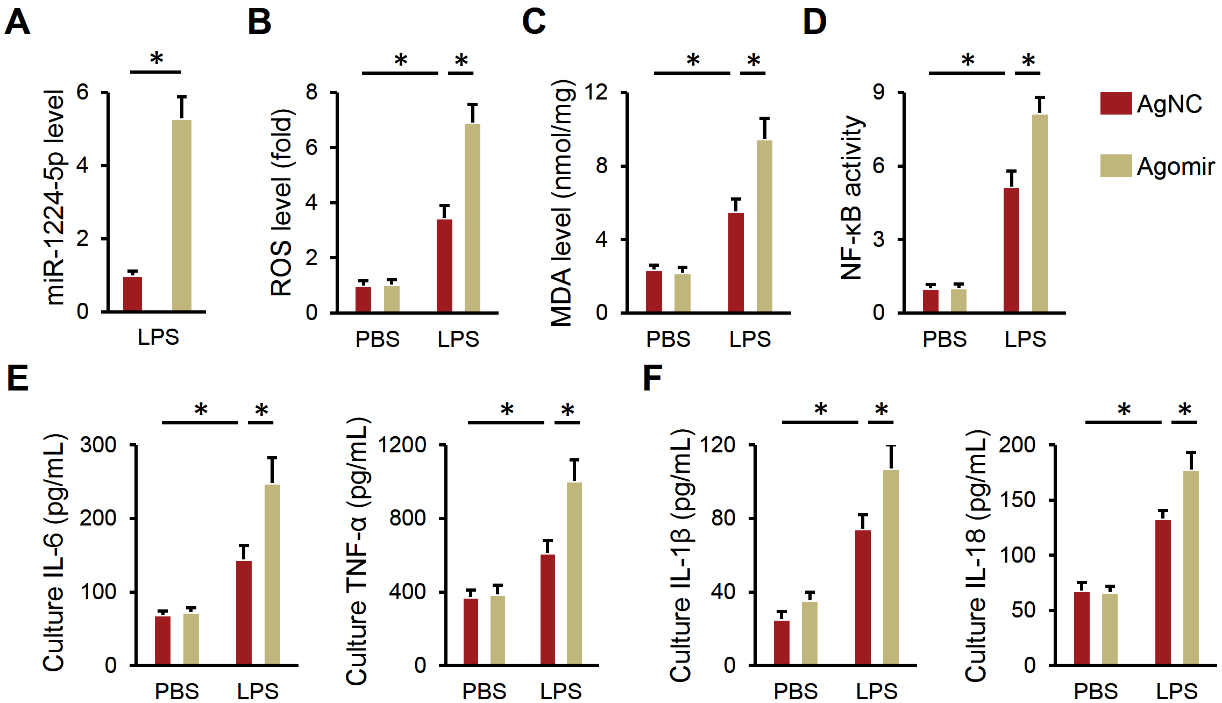


**Figure S2. The miR-1224-5p agomir aggravates oxidative stress and inflammation in LPS-stimulated macrophages. (A)** Macrophages were pre-treated with the miR-1224-5p agomir (50 nmol/L) for 24 h, cultured in fresh medium for an additional 24 h, and then stimulated with or without LPS (100 ng/mL) for 6 h. The levels of miR-1224-5p were detected. **(B)** ROS levels in macrophages were measured by a DCFH-DA probe. **(C)** MDA generations in macrophages. **(D)** NF-κB activities in macrophages. **(E)** The levels of IL-6 and TNF-α in the culture of macrophages. **(F)** The levels of IL-1β and IL-18 in the culture of macrophages. Data represent the means ± SD (n = 6 per group). **P* < 0.05 versus the matched group.


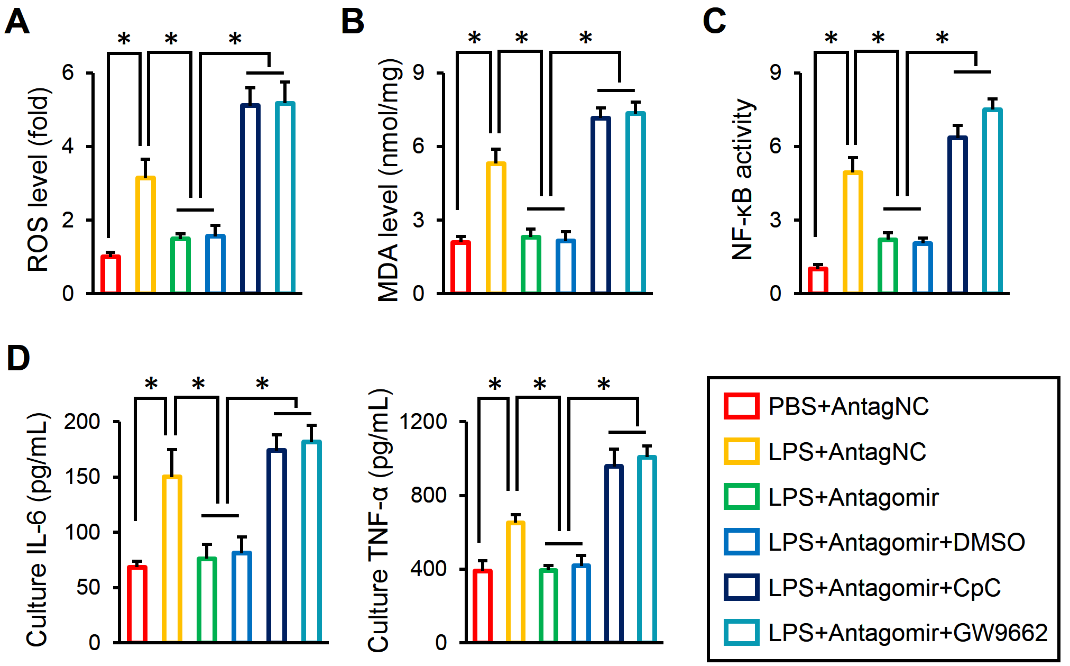


**Figure S3. miR-1224-5p directly targets PPAR-γ to regulate AMPKα activation in macrophages. (A-B)** Macrophages were pre-treated with the miR-1224-5p agomir (50 nmol/L), antagomir (50 nmol/L) or corresponding controls for 24 h, cultured in fresh medium for an additional 24 h, and then stimulated with or without LPS (100 ng/mL) for 6 h. To inhibit AMPKα or PPAR-γ, cells were pre-incubated with CpC (20 μmol/L) or GW9662 (10 μmol/L) for 12 h before miR-1224-5p antagomir treatment. ROS levels in macrophages were measured by a DCFH-DA probe. **(B)** MDA generations in macrophages. **(C)** NF-κB activities in macrophages. **(D)** The levels of IL-6 and TNF-α in the culture of macrophages. Data represent the means ± SD (n = 6 per group). **P* < 0.05 versus the matched group.
